# Supplementary material for: Antiproliferative S-Trityl-l-Cysteine -Derived Compounds as SIRT2 Inhibitors: Repurposing and Solubility Enhancement
Source: Molecules. 2019 Sep 10;24(18):3295. doi: 10.3390/molecules24183295 (PMC6766826; doi:10.3390/molecules24183295)
Supplement: Supplementary file 1 [file molecules-24-03295-s001.pdf]

molecules

Supplementary Material: NMR Data

Antiproliferative *S*-Trityl-L-Cysteine Derived Compounds as SIRT2 Inhibitors: Repurposing and Solubility Enhancement

Mohamed O. Radwan, Halil I. Ciftci, Taha F. S. Ali, Doha E. Ellakwa, Ryoko Koga, Hiroshi Tateishi, Akiko Nakata, Akihiro Ito, Minoru Yoshida, Yoshinari Okamoto, Mikako Fujita* and Masami Otsuka*

* Correspondence: mfujita@kumamoto-u.ac.jp (M.F.); motsuka@gpo.kumamoto-u.ac.jp (M.O.)
Tel.: +81-096-371- 4622 (M.F. & M.O.)

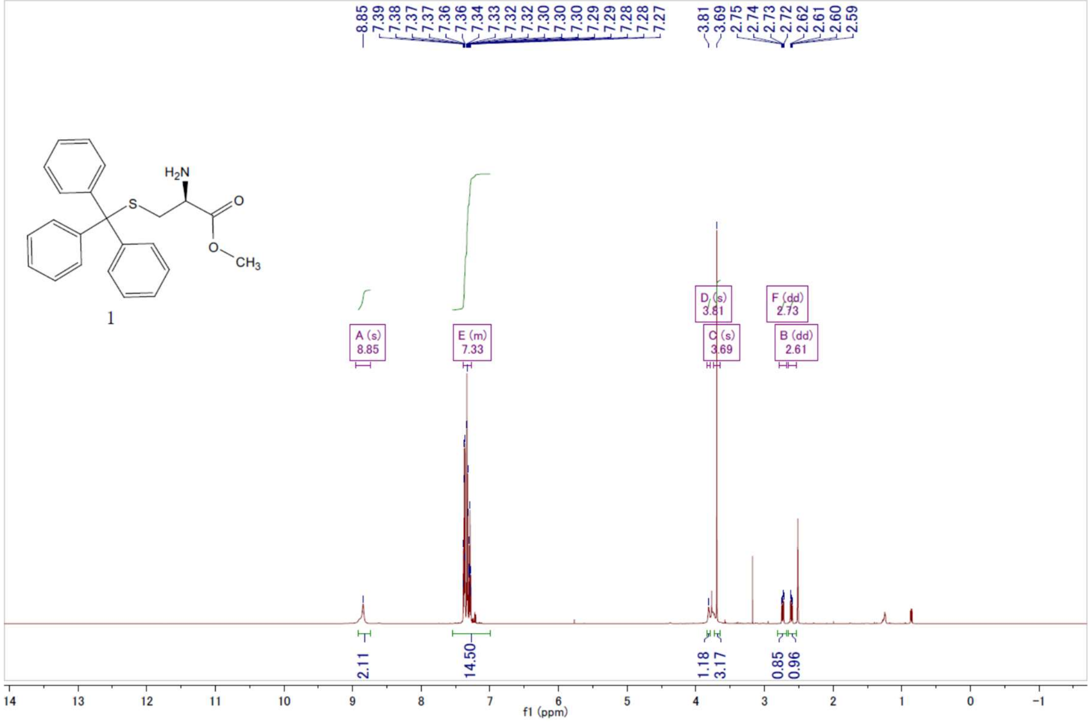

Figure 1. ^{1}H-NMR of compound 1.

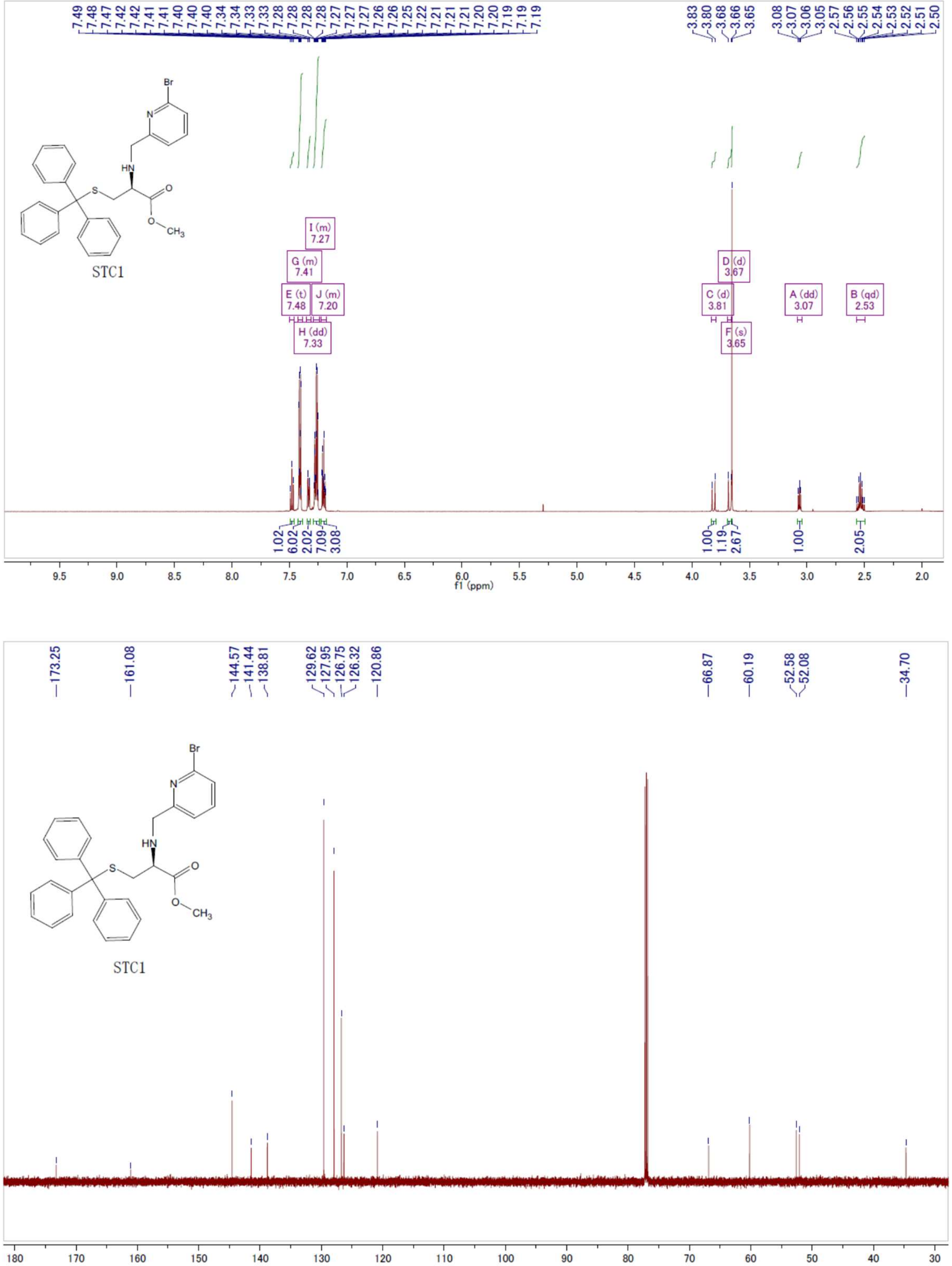

Figure 2. ^{1}H- and ^{13}C-NMR of STC1.

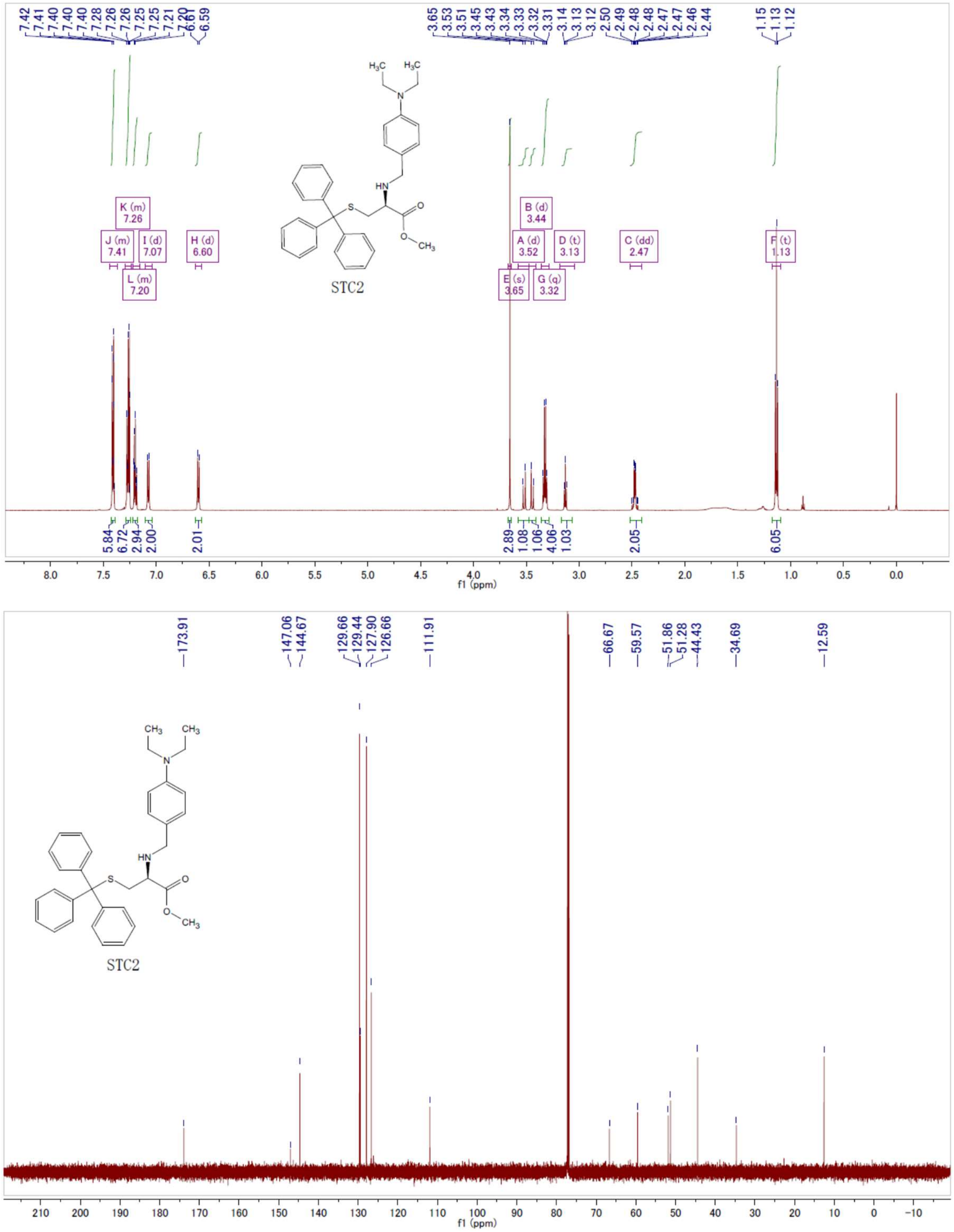

Figure 3. ^{1}H- and ^{13}C-NMR of STC2.

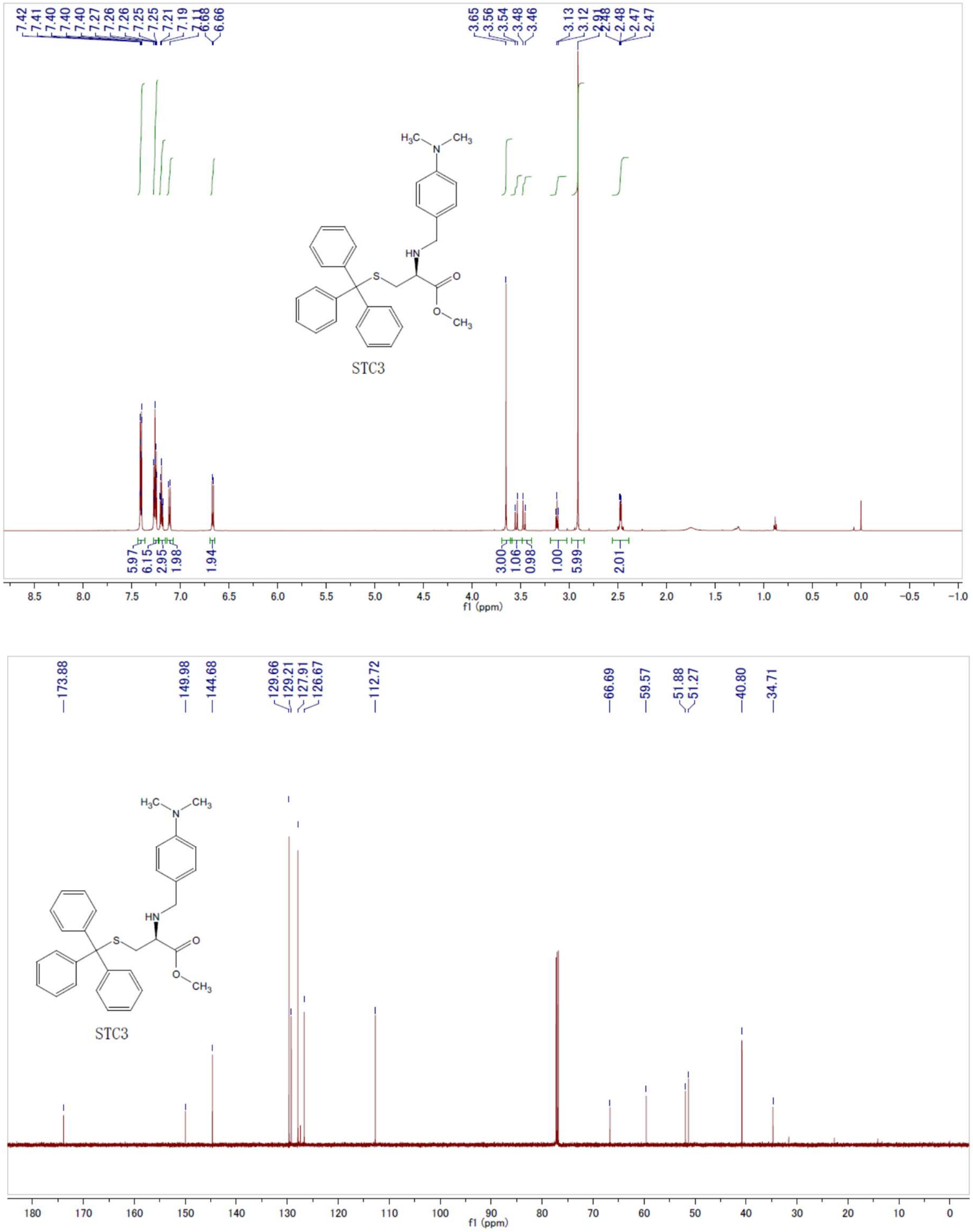

Figure 4. ^{1}H- and ^{13}C-NMR of STC3.

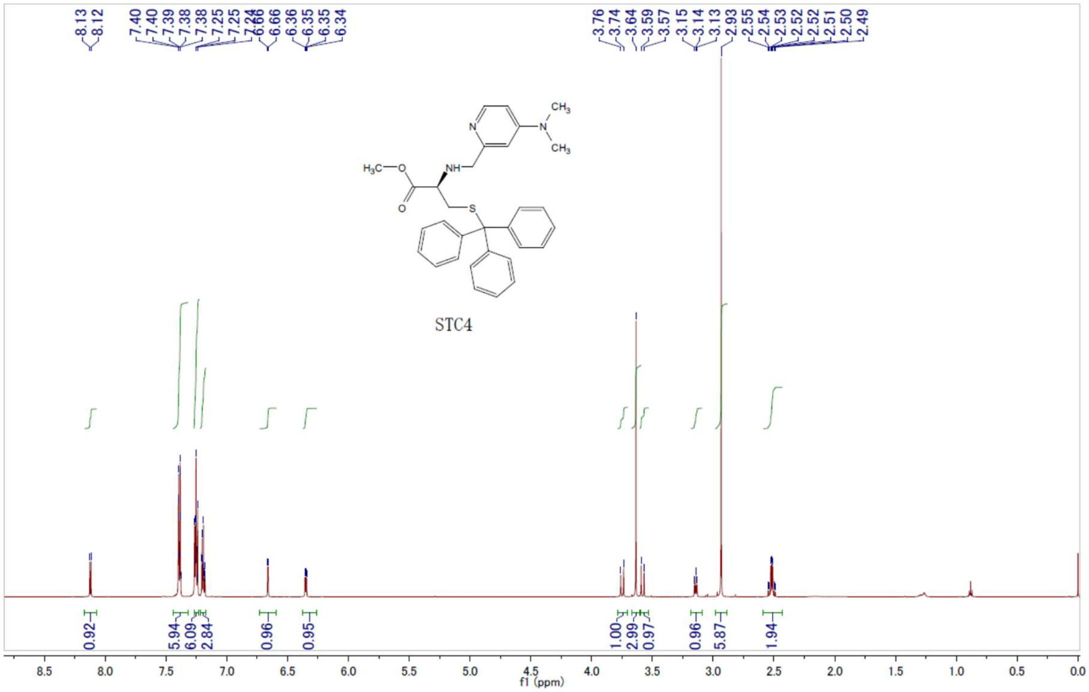

Figure 5. ^{1}H- and ^{13}C-NMR of STC4.

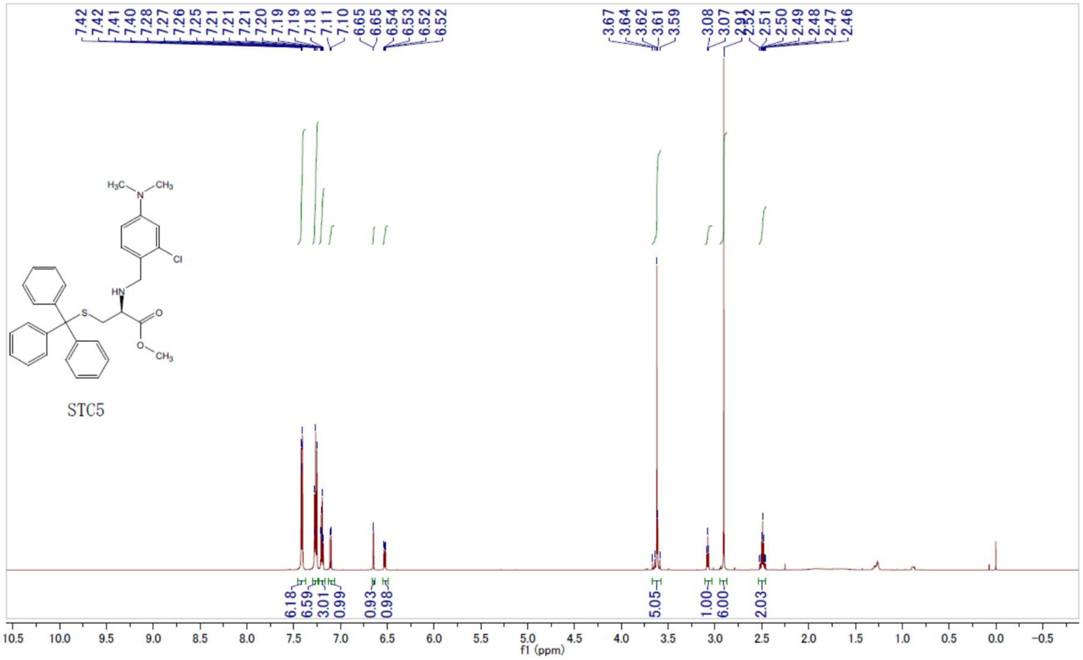

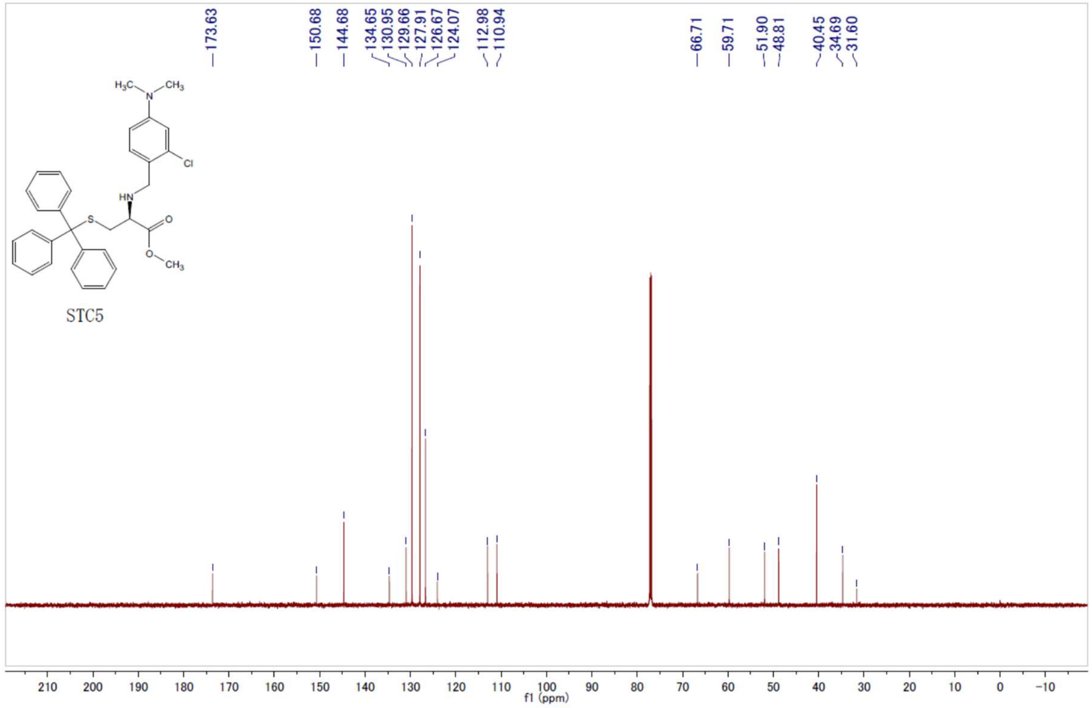

Figure 6. ¹H- and ¹³C-NMR of STC5.

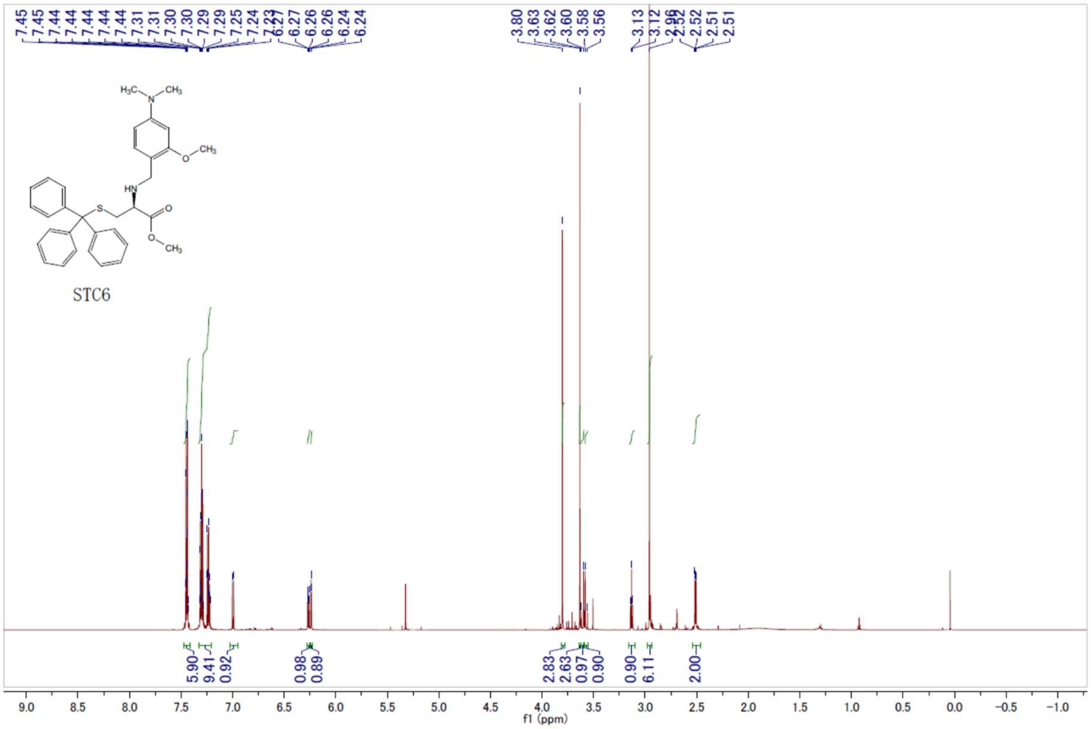

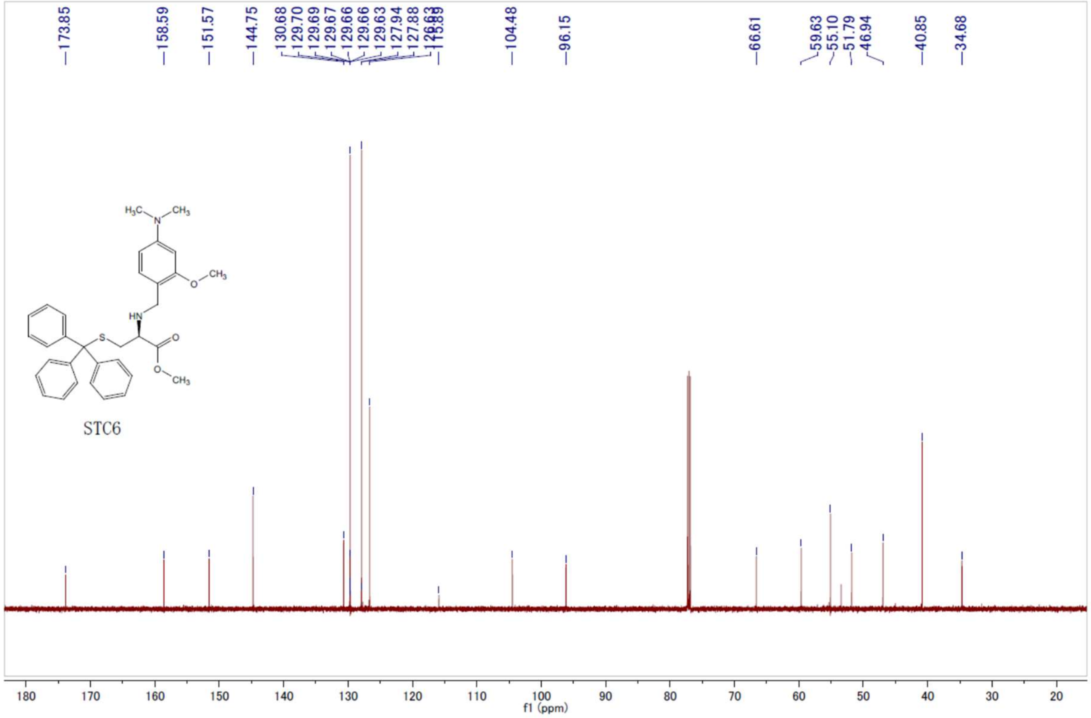

Figure 7. ^{1}H- and ^{13}C-NMR of STC6.

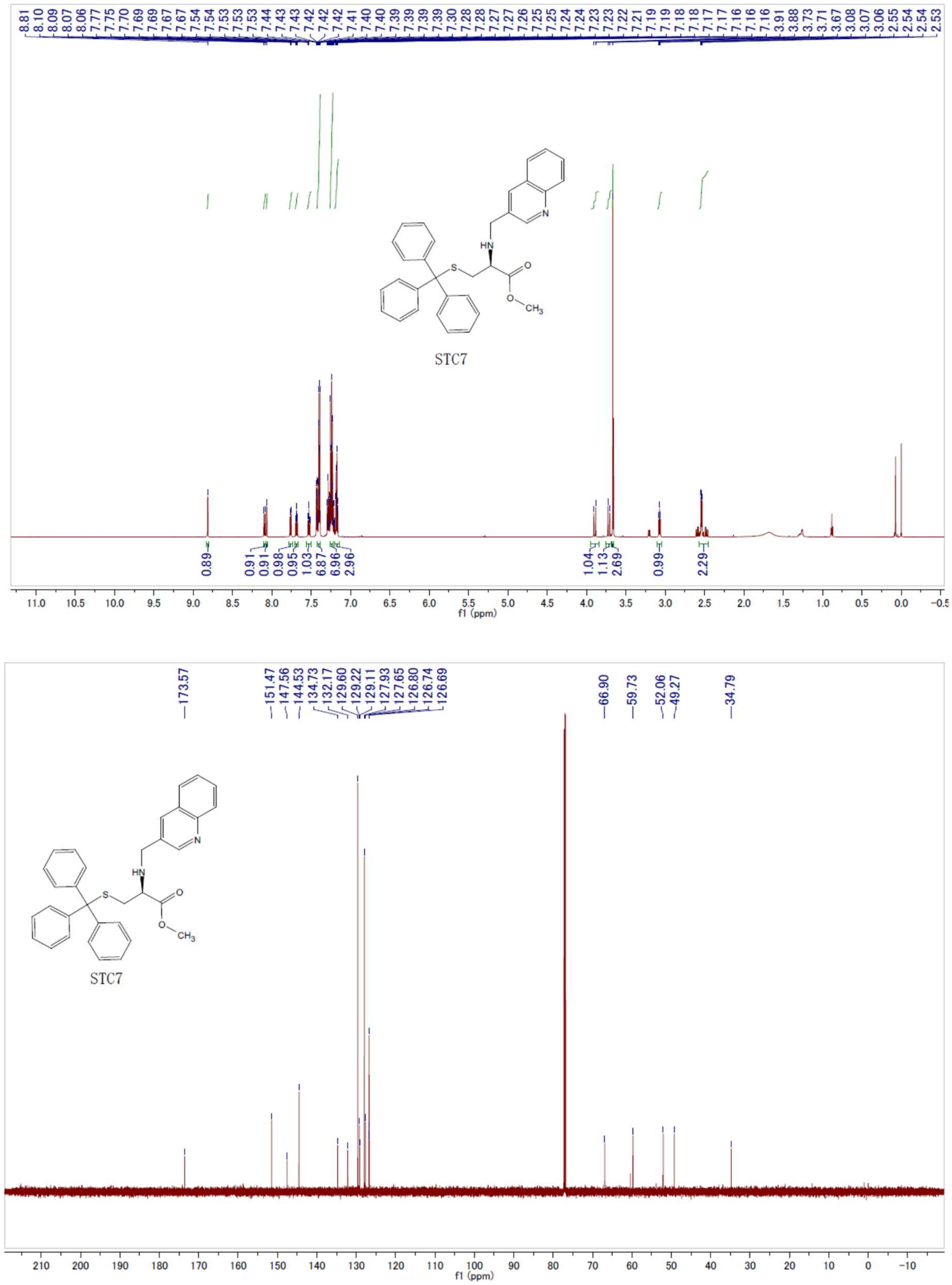

Figure 8. ^{1}H- and ^{13}C-NMR of STC7.

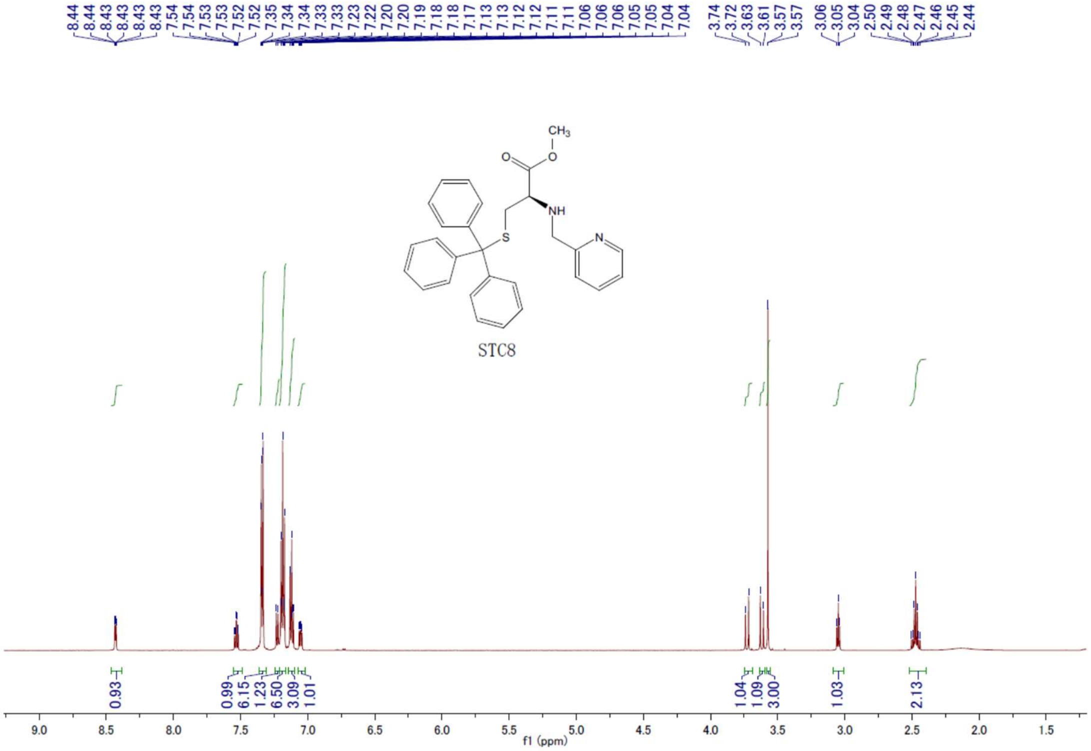

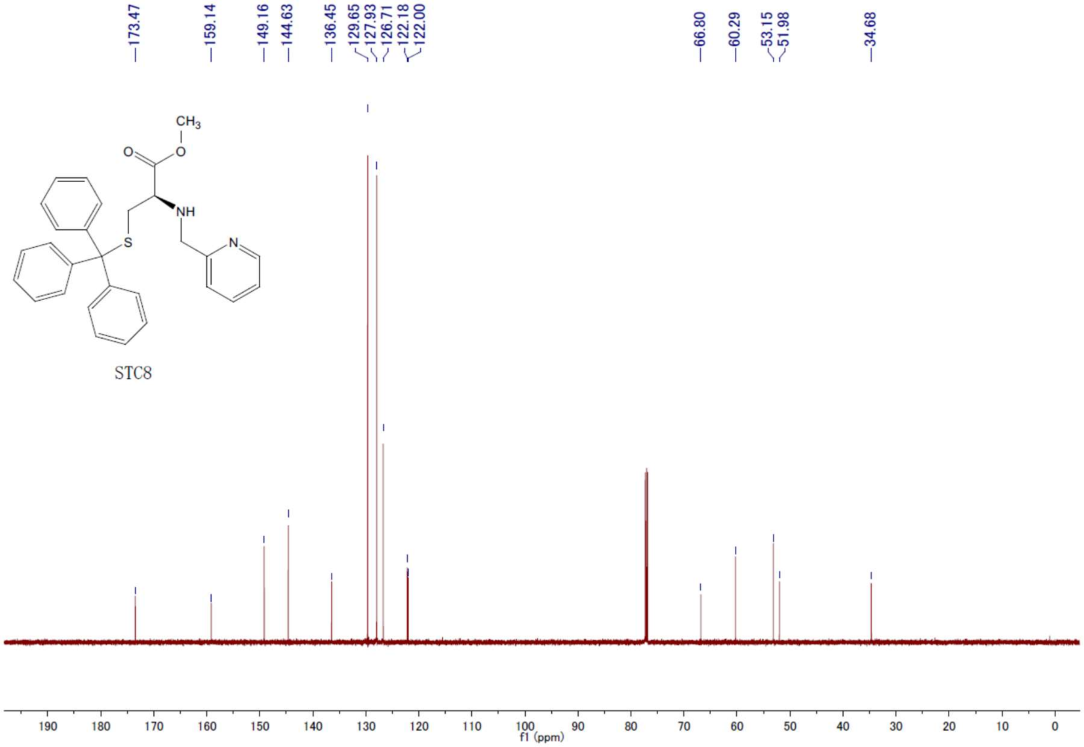

Figure 9. ^{1}H- and ^{13}C-NMR of STC8.

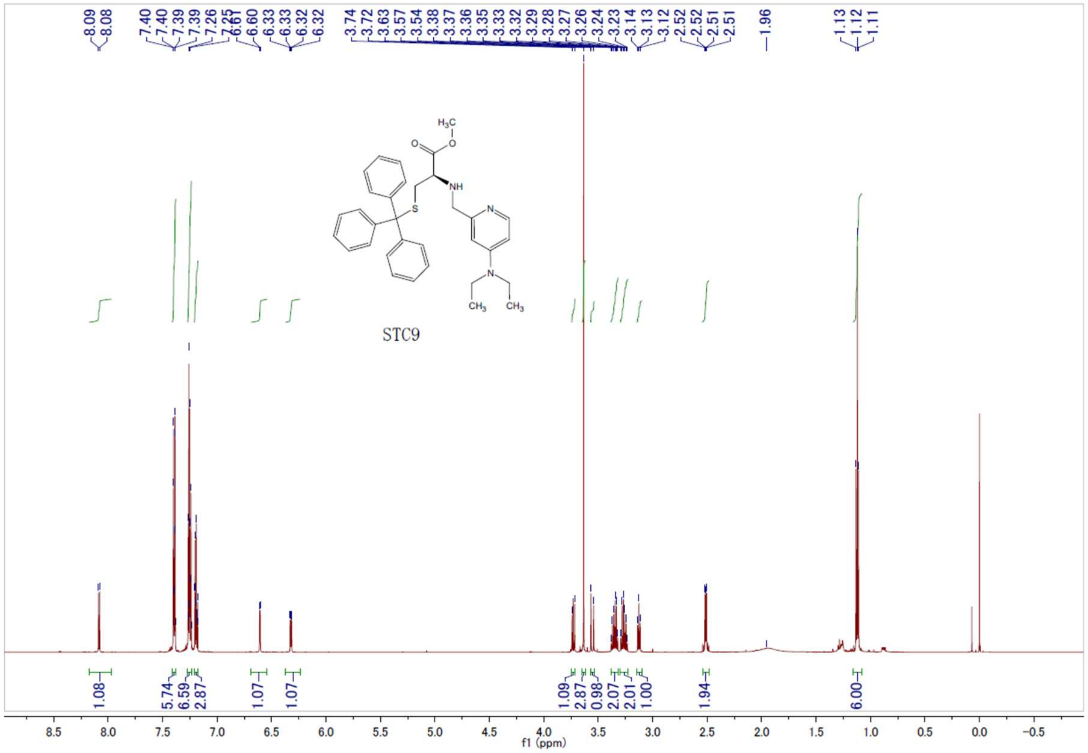

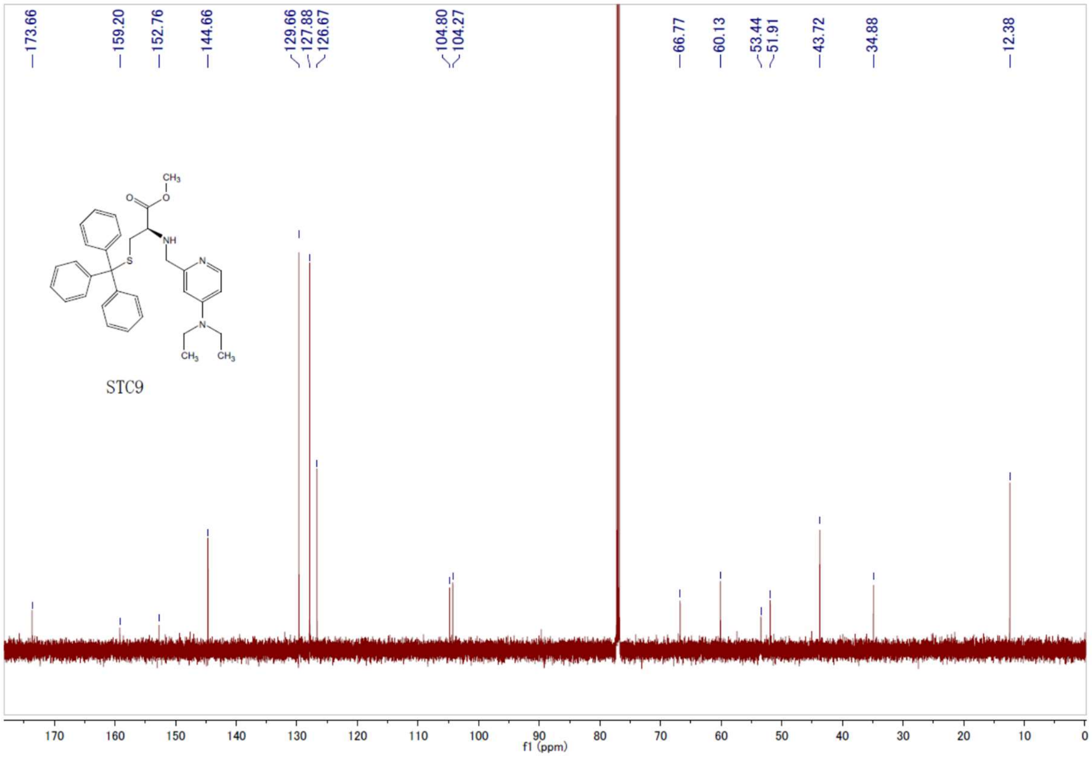

Figure 10. ^{1}H- and ^{13}C-NMR of STC9.

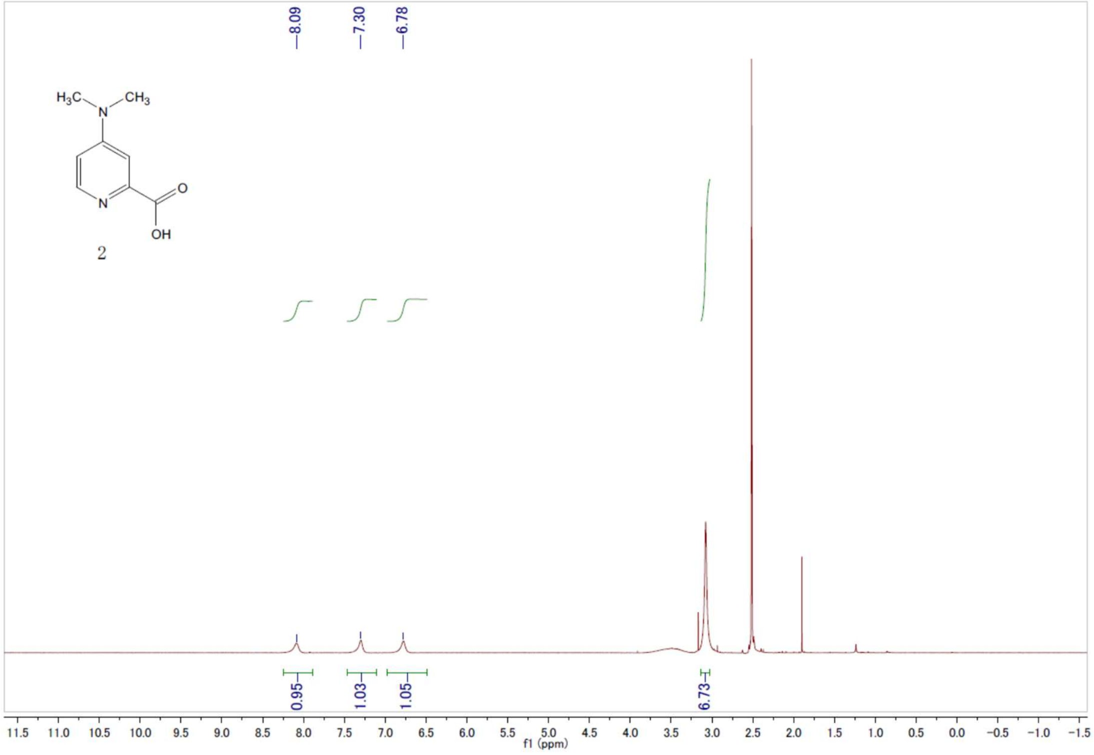

Figure 11. ^{1}H-NMR of compound 2.

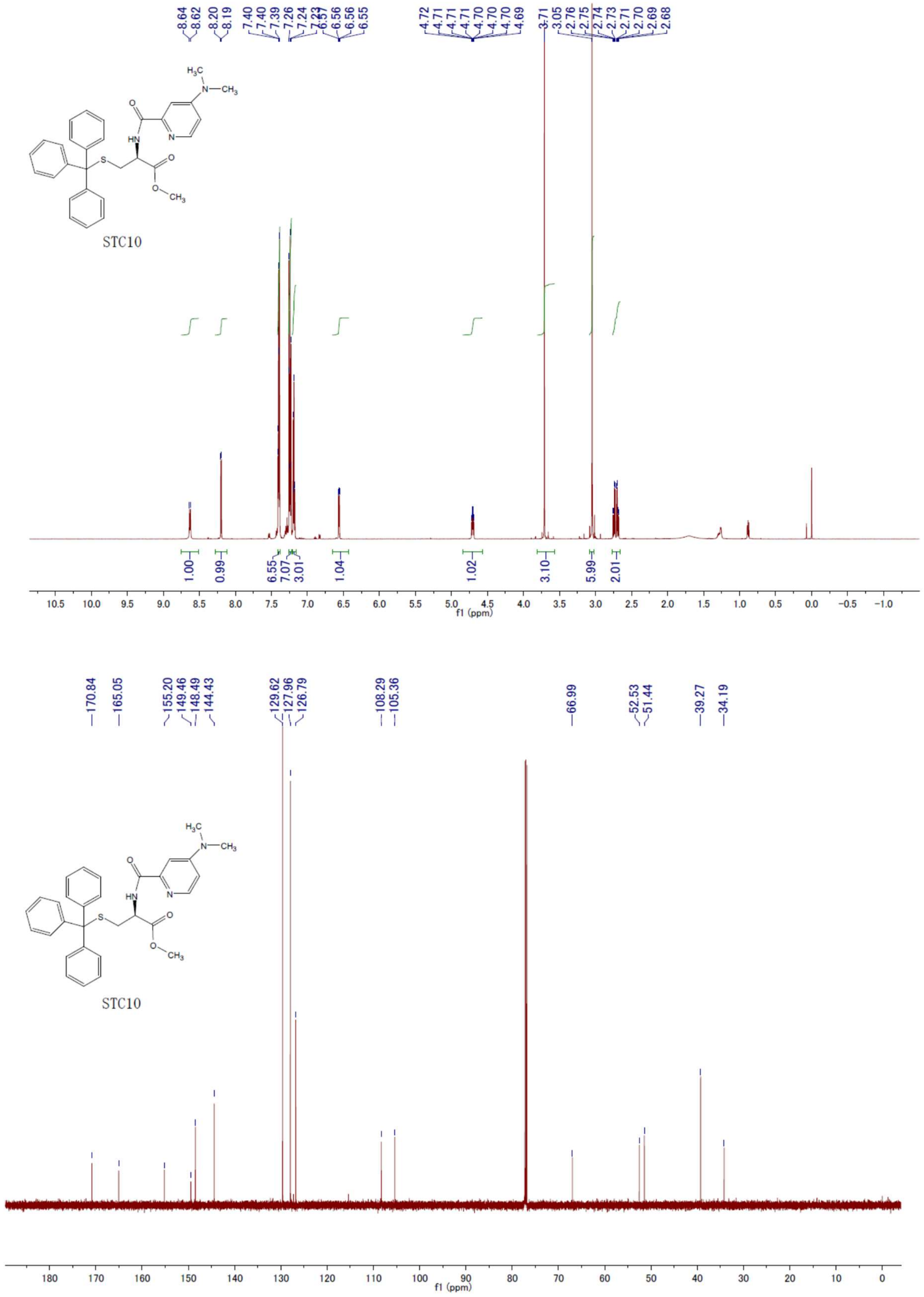

Figure 12. ^{1}H- and ^{13}C-NMR of STC10.

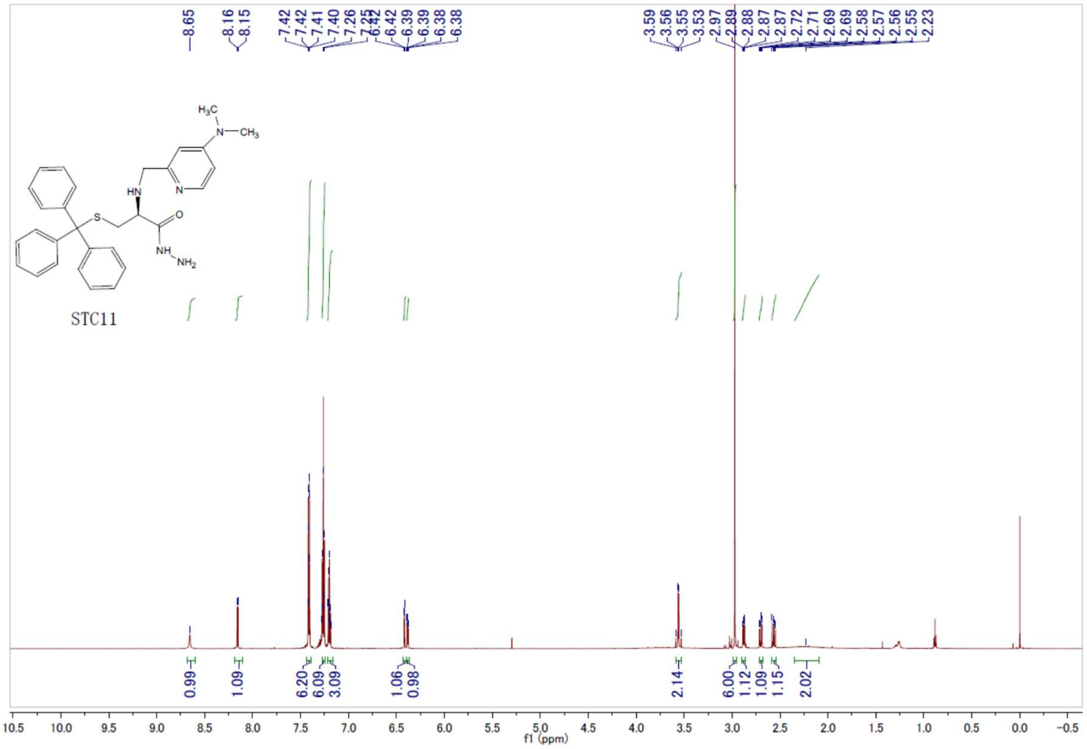

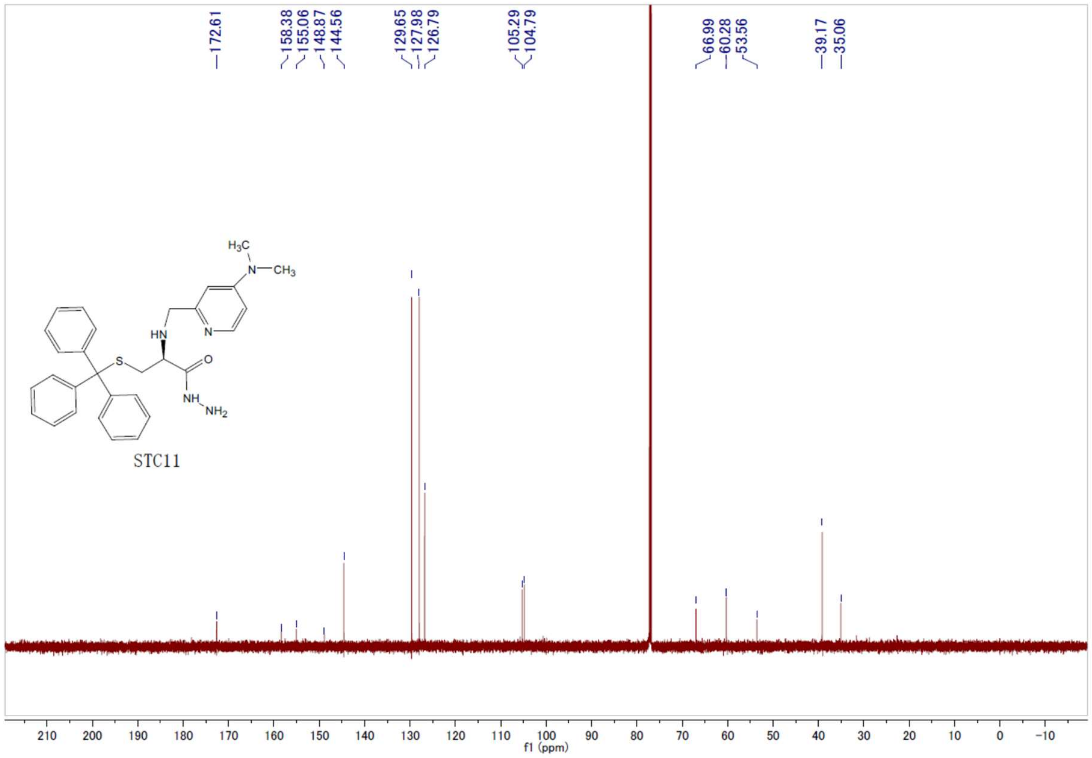

Figure 13. ^{1}H- and ^{13}C-NMR of STC11.
